# Bone marrow stromal cell-conditioned medium regenerates injured sciatic nerve by increasing expression of MPZ and NGF and decreasing apoptosis

**DOI:** 10.22038/IJBMS.2024.74267.16133

**Published:** 2024

**Authors:** Mitra Ghasemi, Athar Talebi, Ali Ghanbari, Parisa Hayat, Behpour Yousefi, Moslem Mohammadi, Mahmood Abedinzade, Nooshin Ahmadirad, Sam Zarbakhsh

**Affiliations:** 1 Nervous System Stem Cells Research Center, Semnan University of Medical Sciences, Semnan, Iran; 2 Department of Anatomy, Faculty of Medicine, Semnan University of Medical Sciences, Semnan, Iran; 3 Laboratory of Learning and Memory, Research Center of Physiology, Semnan University of Medical Sciences, Semnan, Iran; 4 Cellular and Molecular Research Center, Iran University of Medical Sciences, Tehran, Iran; 5 Department of Physiology, Molecular and Cell Biology Research Center, Faculty of Medicine, Mazandaran University of Medical Sciences, Sari, Iran; 6 Medical Biotechnology Research Center, Department of Physiology, School of Paramedicine, Guilan University of Medical Sciences, Rasht, Iran

**Keywords:** Apoptosis, Conditioned medium, Mesenchymal stem cells, Myelin protein zero, Nerve growth factor, Sciatic nerve

## Abstract

**Objective(s):**

Despite the many benefits of mesenchymal stem cell (MSC) transplantation for tissue regeneration, there are some limitations to using them, including the high costs, applying invasive procedures, the possibility of transplant rejection, and cell malignancy. This study aimed to investigate the effect of secretions of bone marrow stromal cells (BMSCs) with the cell-free strategy on damaged sciatic nerve with an emphasis on the role of apoptosis and the expression of myelin protein zero (MPZ) and nerve growth factor (NGF) proteins.

**Materials and Methods:**

BMSCs were cultured and a 25-fold concentrated conditioned medium (CM) from the cells was provided. After creating a crush injury in the left sciatic nerve of male rats, BMSCs or CM were injected into the injured site of the nerve. Four weeks later, the expression of MPZ, NGF, Bax, and Bcl-2 proteins in the sciatic nerve and histological parameters of the sciatic nerve and gastrocnemius muscle were assessed.

**Results:**

The results demonstrated that injection of CM decreased apoptosis and increased expression of MPZ and NGF proteins, improving remyelination and regeneration of the sciatic nerve almost as much as the transplantation of the BMSCs themselves compared to the control group.

**Conclusion:**

The results suggest that BMSC secretions may improve remyelination and regeneration of damaged sciatic nerve by increasing the expression of MPZ and NGF and decreasing apoptosis.

## Introduction

Peripheral nerve damage is relatively common, limiting activity, causing psychological and physiological problems, and, if healing is delayed, leads to irreversible muscle atrophy and fibrosis (1). In this type of injury, destruction of axons and myelin occurs in the proximal and distal parts of the nerve from the site of injury. The distal axons are degraded and gradually disappear. The proximal axons begin to respond and regenerate during which axonal growth occurs toward the distal segment (2). Methods that can cause more growth of axons produce more favorable results (3).

Myelin protein zero (MPZ) and nerve growth factor (NGF) are two proteins produced by Schwann cells. When peripheral nerves are injured, the expression of these proteins increases, leading to myelination, growth, and repair of axons. MPZ is the main protein for myelination in peripheral nerves, and NGF is an important neurotrophic growth factor that stimulates axon growth (4, 5).

One of the methods that has recently been considered for the repair of injured peripheral nerves is the transplantation of mesenchymal stem cells (MSCs) to the nerve. The types of mesenchymal stem cells most commonly used to repair damaged peripheral nerves are bone marrow stromal cells (BMSCs), adipose-derived stem cells, and umbilical cord stromal cells (6, 7). Mesenchymal stem cells can migrate to damaged tissues, differentiate into other cell lines, and produce different growth factors such as NGF, brain-derived neurotrophic factor (BDNF), and vascular endothelial growth factor (VEGF) (8, 9). It seems that the greatest therapeutic effect of MSCs on damaged tissues is due to their ability to produce growth factors, not to differentiate into other cell types (10, 11). 

Despite the many benefits of MSC transplantation for tissue regeneration, there are limitations to using them, including the high costs, the application of invasive procedures, the poor survival rate of MSCs during transplantation, the possibility of transplant rejection, and cell malignancy (12, 13). A novel idea with the cell-free strategy for improving damaged tissues is to use the secretions of MSCs rather than the cells themselves, given that the secretions of MSCs are probably responsible for a significant part of the therapeutic potential of these cells (9).

In the current study, the impact of transplantation of BMSCs and BMSC secretions without the cells themselves on the expression of MPZ and NGF proteins, apoptosis, remyelination, and regeneration of the injured sciatic nerve was evaluated in rats.

## Materials and Methods

***Experimental animals***

In this study, 32 adult male Wistar rats (250–300 g) were used, which had free access to food and water under controlled temperatures (25 ± 2 °C). All experimental procedures were approved by the ethical committee of Semnan University of Medical Sciences (Semnan, Iran). The ethical code is IR.SEMUMS.AEC.1401.002.

***Bone marrow stromal cell culture***

Bone marrow was ejected from the femurs and tibias of an adult rat and cultured in Dulbecco’s Modified Eagle Medium (DMEM) (Gibco, Germany) containing 10% fetal bovine serum (FBS) (Gibco, Germany) and 1% pen/strep (Gibco, Germany), incubated at 37 °C, 95% humidity, and 5% CO_2_. The cells were subcultured four times (14, 15). 

***Analysis of the cell surface markers***

To analyze the expression of the surface markers of BMSCs, the cells were incubated with fluorescence-labeled monoclonal antibodies conjugated with fluorescein isothiocyanate (FITC) against CD29, CD34, CD44, CD45, and CD90 (Sigma, China). Then the labeled cells were analyzed with a BD FACS Calibur flow cytometry apparatus (16).

***Preparation of conditioned medium (CM)***

About 1×10^6^ BMSCs at passage four were re-cultured in 1 ml DMEM with serum-free medium for 24 hr. Then, the medium was concentrated 25-fold using ultrafiltration units with a 5 kDa molecular weight cut-off (Amicon, Millipore, USA) (13).

***Creating the model of crush injury***

To create the sciatic nerve crush injury model, the rats were anesthetized by intraperitoneal injection of 80 mg/kg ketamine and 10 mg/kg xylazine, and the left sciatic nerve was exposed. Crush injury was accomplished 1 cm below the sciatic notch by clamping with non-serrated hemostatic forceps for 30 sec. So that the nerve was cut entirely while the epineurium was preserved. Then the nerve was gently returned to its place and the skin was sutured with 4-0 silk (17).

***Animal groups***

The rats were randomly divided into four groups: Sham, control, BMSCs, and conditioned medium (CM) (n=8 in each). In the sham group, the left sciatic nerve was exposed and repositioned to its place without crush injury. In the groups of control, BMSCs, and CM, after creating the nerve crush injury, using a Hamilton microsyringe, respectively, 10 µl of culture medium, 1×10^6^ BMSCs in 10 µl of culture medium, and 10 µl of the CM were directly injected into the nerve injury site (18, 19).

***Western blot assays***

Four weeks after surgery, the expression of MPZ, NGF, Bcl-2, and Bax proteins in the sciatic nerves was evaluated by western blotting. Briefly, the nerves were lysed using radioimmunoprecipitation assay (RIPA) buffer (Roche, Switzerland). After separating proteins using electrophoresis, the proteins were transferred to nitrocellulose membranes (Amersham Biosciences, USA) and were blocked with 5% skim milk. Next, the membranes were incubated with primary antibodies for MPZ, NGF, Bcl-2, Bax, and β-actin (1:1000, Abcam, USA) overnight at 4 °C. After incubating membranes with the secondary antibody, goat anti-rabbit conjugated with horseradish peroxidase (HRP) (1:5000, Santa Cruz Biotechnology, USA), immunoreactive bands were demonstrated with a chemiluminescence detection kit (Amersham Biosciences, USA) and quantified with ImageJ software (4, 20).

***Histological evaluation of the sciatic nerve***

Four weeks after surgery, to assess the histological indices of the sciatic nerve, the nerve was fixed in 2.5% glutaraldehyde and post-fixed in 2% osmium tetroxide. After embedding in Epon resin, semi-thin (700 nm) and ultra-thin (70 nm) sections were obtained using an ultramicrotome. Semi-thin sections were stained with toluidine blue to measure the nerve fiber area/µm^2^ and nerve fiber diameter/µm by an Olympus light microscope, and ultra-thin sections were prepared to measure myelin sheath thickness/µm by a TEM (Zeiss EM 10 CR TEM, Jenna, Germany). The measurements were done using the ImageJ software package (21). 

***Histological evaluation of the gastrocnemius muscle***

Four weeks after surgery, to evaluate the histology of the gastrocnemius muscle, the left muscle was removed immediately after the removal of the left sciatic nerve and fixed in 10% formalin. After processing, 5 µm thick sections were provided. Five sections from each muscle were randomly selected and stained with hematoxylin and eosin (H&E). Finally, to assess the amount of muscle atrophy, cross-sectional muscle fiber area/mm^2^ was evaluated by ImageJ software (22). 

***Statistical analyses***

Statistical analyses were done with the one-way analysis of variance (ANOVA) followed by the Tukey test. The data were presented as mean ± SE, and a level of *P*<0.05 was considered statistically significant.

***BMSC culture and characterization***

**Figure 1:**
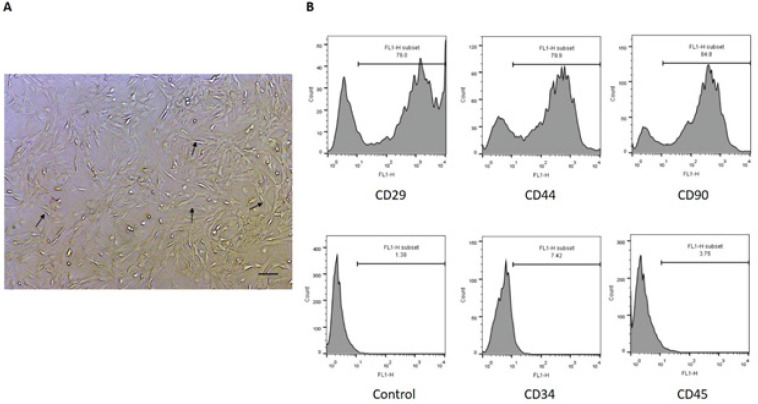
Cultured rat bone marrow stromal cells (BMSCs) at Passage 3 (A). Results of flow cytometry showed that BMSCs expressed CD29, CD44, and CD90 and did not express CD34 and CD45 (B). Arrows indicate spindle-shaped cells with elongated nuclei

The cultured BMSCs stuck to the bottom of the flask and resembled spindle-shaped fibroblast cells (Figure 1A). Most of the cells expressed the markers of the stromal cells including CD29, CD44, and CD90 while they did not express hematopoietic markers including CD34 and CD45 (Figure 1B).

***Western blot assays***

**Figure 2:**
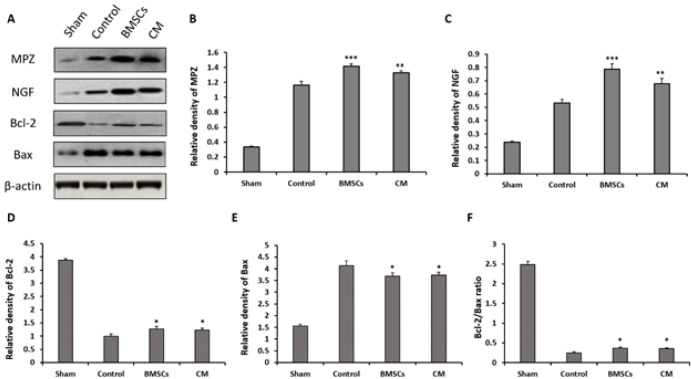
Four weeks after surgery, the effects of bone marrow stromal cells (BMSCs) and conditioned medium (CM) on the expression of myelin protein zero (MPZ), nerve growth factor (NGF), Bcl-2 and Bax proteins in damaged rat sciatic nerves were measured using Western blot

Western blot assays were done to assess the expression level of MPZ and NGF proteins and to assess apoptosis by evaluating the expression level of Bcl-2 and Bax proteins in the sciatic nerve. The results of the expression of MPZ and NGF proteins demonstrated that transplantation of BMSCs and CM significantly enhanced the expression of MPZ (*P*<0.001 and *P*=0.007, respectively) and NGF (*P*<0.001 and *P*=0.003, respectively) proteins compared to the control group. Also, the expression of NGF in the BMSC group was significantly higher than in the CM group (*P*=0.005), but there was no statistically significant difference in the expression of MPZ between BMSC and CM groups (*P*=0.147) (Figure 2).

The results of apoptosis demonstrated that transplantation of BMSCs and CM significantly increased the expression of Bcl-2 protein (*P*=0.011 and *P*=0.024, respectively) and decreased the expression of Bax protein (*P*=0.021 and *P*=0.038, respectively) compared to the control group. There was no statistically significant difference in the expression of Bcl-2 and Bax proteins between the BMSC and CM groups (*P*=0.912 and *P*=0.973, respectively). Bcl-2/Bax expression ratio enhanced in both BMSC and CM groups compared to the control group (*P*=0.038 and *P*=0.048, respectively) but there was no significant difference between BMSC and CM groups (*P*=0.998) (Figure 2).

***Histological assessment of the sciatic nerve***

**Figure 3:**
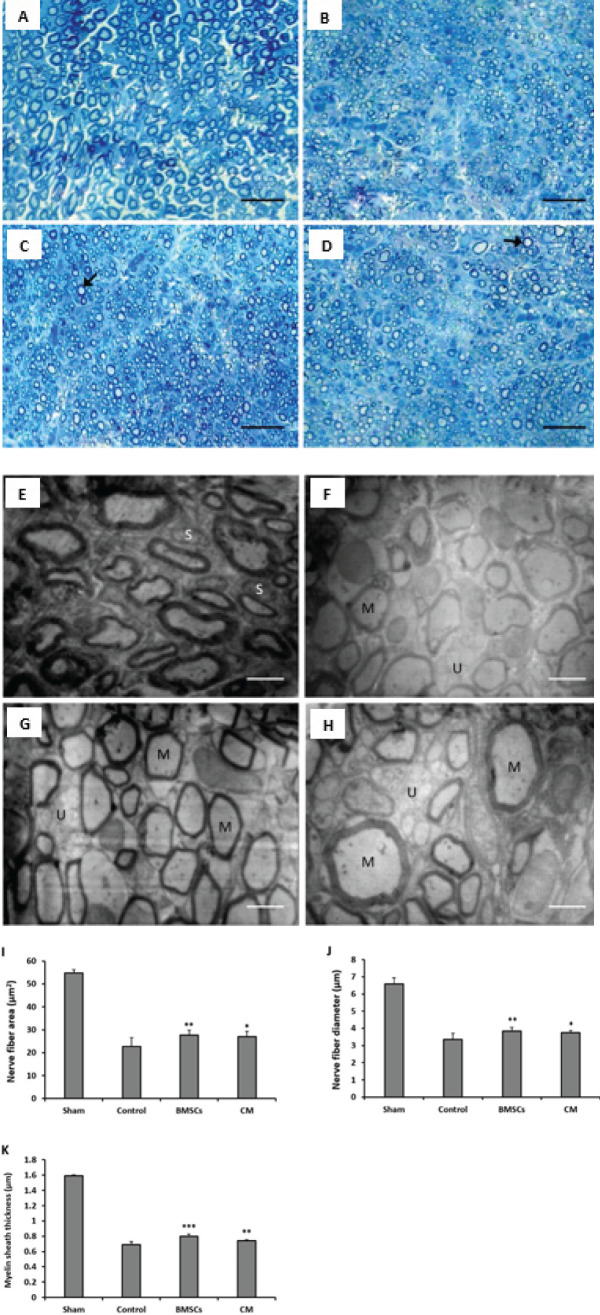
Four weeks after surgery, the effects of bone marrow stromal cells (BMSCs) and conditioned medium (CM) on the histological indices of the damaged rat sciatic nerves were measured. Toluidine blue staining (A-D) to calculate the mean of area and diameter of nerve fibers and transmission electron microscope images (E-H) to calculate the mean of myelin sheath thickness of nerve fibers from the cross-sections of sciatic nerves in sham (A, E), control (B, F), BMSC (C, G), and CM (D, H) groups. The results of nerve fiber area (I), nerve fiber diameter (J), and myelin sheath thickness (K). Well-compacted myelinated nerve fibers (arrows), Myelinated axons (M), unmyelinated axons (U), and Schwann cells (S) are indicated. Scale bars: 50 μm (A-D) and 5 µm (E-H)

The amount of sciatic nerve regeneration was evaluated by calculating the histological parameters of the nerve using light and transmission electron microscopes. The results showed that the crush injury (control group) significantly decreased the histomorphometric parameters of the sciatic nerve compared to the sham group (*P*<0.001). Transplantation of BMSCs and CM significantly increased the histomorphometric parameters including the nerve fiber area (*P*=0.003 and *P*=0.011, respectively), nerve fiber diameter (*P*=0.008 and *P*=0.045, respectively), and myelin sheath thickness (*P*<0.001 and *P*=0.005, respectively) compared to the control group. There was no significant difference in nerve fiber area, nerve fiber diameter, and myelin sheath thickness of sciatic nerve between BMSC and CM groups (*P*=0.953, *P*=0.889, and *P*=0.56, respectively) (Figure 3).

***Histological assessment of the gastrocnemius muscle***

**Figure 4:**
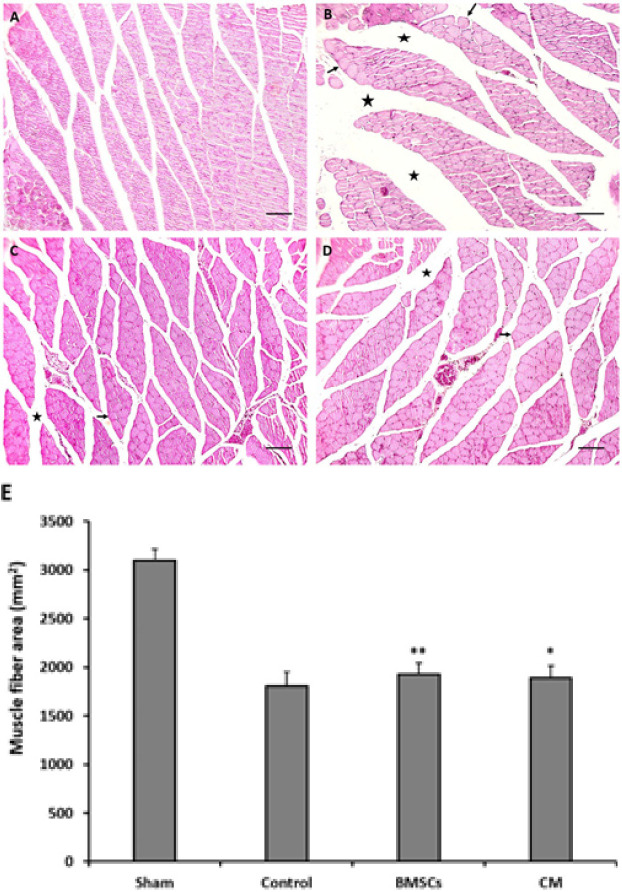
Effects of bone marrow stromal cells (BMSCs) and conditioned medium (CM) on gastrocnemius muscle fiber area four weeks after rat sciatic nerve injury

The amount of gastrocnemius muscle regeneration was evaluated by measuring the muscle fiber area. The results demonstrated that four weeks after the crush injury (control group), the muscle fiber area significantly reduced compared to the sham group (*P*<0.001), while transplantation of BMSCs and CM significantly increased the muscle fiber area compared to the control group (*P*=0.002 and *P*=0.043, respectively). There was no statistically significant difference between BMSC and CM groups (*P*=0.604) (Figure 4).

Scale bar: 100 µm.

Immunoblot of MPZ, NGF, Bcl-2, Bax, and β-actin proteins (A). Results of expression of MPZ (B), NGF (C), Bcl-2 (D), and Bax (E) proteins. The results of the expression ratio of Bcl-2/Bax proteins (F). *P<0.05, **P<0.01, ***P<0.001 vs the control group.

*P<0.05, **P<0.01, ***P<0.001 vs the control group.

Cross-sections of the muscles with hematoxylin and eosin (H&E) staining are indicated in sham (A), control (B), BMSC (C), and CM (D) groups. The results of the mean of muscle fiber area (E). Arrows show the muscle fibers. Stars in histological figures show the connective tissue between muscle fibers, the more it is, the more atrophy has occurred. Scale bars: 100 µm. *P<0.05, **P<0.01 vs the control group.

## Discussion

Various studies indicate that BMSC transplantation can improve the repair process in damaged peripheral nerves (6, 23). However, stem cell transplantation has limitations such as high costs, invasive procedures, the possibility of transplant rejection, and malignancy that make it difficult to use (9). One alternative is probably to use the secretions of these cells without transplanting the cells themselves (13). In the current study, we investigated the effect of transplantation of BMSCs and their secretions collected in the conditioned medium (CM) on the rat-damaged sciatic nerve with an emphasis on the role of apoptosis and the expression of MPZ and NGF proteins which are associated with remyelination and nerve growth. In general, the results showed that CM transplantation significantly improved remyelination and regeneration of damaged peripheral nerves and could possibly be a suitable alternative to BMSC transplantation.

MSCs secrete a variety of peptides, proteins, and lipid mediators that can be frozen and concentrated without loss of activity (24). Some studies have demonstrated that the greatest effect of MSCs transplantation is due to their ability to secrete growth factors such as NGF, BDNF, and VEGF which have a beneficial effect on damaged tissues, not their ability to differentiate into other cells (9-11). Some reports have shown that the factors secreted by stem cells in the absence of the cells may cause the regeneration of damaged tissues (25, 26). Factors secreted from stem cells that are released in the cell culture medium are known as secretomes, so this medium is called the conditioned medium (CM) (13).

In the present study, four weeks after the sciatic nerve crush injury, the expression of MPZ and NGF proteins in the nerve was evaluated with western blot. MPZ is the main protein expressed in the peripheral nerve myelin sheath which is produced by Schwann cells. The expression of MPZ has a direct relationship with remyelination (27, 28). NGF is one of the important neurotrophic factors that can stimulate the growth and lengthening of axons and nerve regeneration (29). Normally, the expression of MPZ and NGF proteins is very low. At the time of peripheral nerve damage, the expression level of these proteins increases suddenly (4). The results demonstrated that both BMSCs and CM could enhance the expression of these proteins in the injured sciatic nerve compared to the control group. The results of BMSC transplantation showed that BMSCs could induce the expression of MPZ and NGF which were consistent with other studies. Chen *et al*. have shown that transplantation of BMSCs repairs peripheral nerves by secreting essential growth factors such as BDNF, NGF, glial cell line-derived neurotrophic factor (GDNF), and ciliary neurotrophic factor (CNTF) (30). An *in vitro* study demonstrated that BMSCs caused MPZ expression as an indicator of Schwann cells (31). Moreover, Mathot *et al*. reported that transplantation of MSCs leads to a significant up-regulation of the expression of numerous genes such as MPZ and NGF, which are important for peripheral nerve regeneration over time (32). The reasons why BMSCs were able to increase the expression of MPZ and NGF were probably due to the production of necessary neurotrophic factors and stimulation of Schwann cells to produce MPZ and NGF, the differentiation of BMSCs into Schwann cells, and the regeneration potential of BMSCs (30-32).

The results of the CM group in the present study showed that CM could also increase the expression of MPZ and NGF proteins. There is no similar study on the effect of BMSC-CM on MPZ expression, but the results of NGF protein expression were consistent with other relevant in vitro studies (33, 34). The comparison results between BMSC and CM groups showed that there was no significant difference in the expression of MPZ between these groups, but the expression of NGF in the BMSC group was significantly higher compared to the CM group, which was probably due to the role of BMSCs in NGF secretion (30). The reasons why CM was able to increase the expression of MPZ and NGF were probably due to the fact that NGF is one of the secretions of MSCs, which increases the survival of cells and increases the expression of neurotrophic factors of Schwann cells like MPZ and NGF. In addition, CM increases Schwann cell proliferation by activating the MAPK / Erk pathway, an important signal transduction pathway that affects gene expression, cell survival, cell proliferation, and cell apoptosis (33). 

To assess the amount of apoptosis in the sciatic nerve, the expression ratio of Bcl-2/Bax proteins was assessed with western blot. The results showed that both BMSCs and CM could increase the expression ratio of Bcl-2/Bax proteins in the injured sciatic nerve compared to the control group, and there were no significant differences between the BMSC and CM groups. BMSCs have immunomodulatory properties that help to decrease oxidative stress, inflammation, and apoptosis in peripheral nerves (35). BMSCs can reduce apoptosis in peripheral nerves by the production of neurotrophic factors, especially NGF, which is an important mediator of protection against neuronal apoptosis (34, 36). NGF reduces neural cell apoptosis by decreasing the expression of caspase-3 and up-regulating the expression of Bcl-2 (37). On the other hand, CM reduces the mitochondria-dependent apoptosis in neurons via the NGF and relies on the Akt/Bad pathway, which is a key regulator for the anti-apoptotic role of CM (34). The results of apoptosis were in agreement with other studies (34, 36).

To evaluate remyelination and regeneration of the sciatic nerve, histological evaluations were performed using light and transmission electron microscopes. Also, histological evaluation of the gastrocnemius muscle was done because repairment of the gastrocnemius muscle is one of the parameters of the regeneration of damaged sciatic nerve. That is, the lower the amount of atrophy in the gastrocnemius muscle, the more the sciatic nerve has been repaired (6). Based on the western blot results, BMSCs and CM probably increased remyelination and regeneration of the nerve by inducing the expression of MPZ and NGF proteins and reducing apoptosis. The results of histology were consistent with other studies. A study showed that MSC-derived extracellular vesicles could promote rat sciatic nerve regeneration after crush injury (38). Other researchers have demonstrated that stem cell secretions of human deciduous teeth regenerate the sciatic nerve and gastrocnemius muscle in rats (39). Another study reported that BMSC-CM regenerated the sciatic nerve and gastrocnemius muscle in rabbits (40). Namini *et al*. have shown that exosomes of human endometrial stem cells regenerate peripheral nerves in rats (41).

Since paracrine growth factors secreted by MSCs can accumulate in the CM, it can be applied as a cell-free therapy. MSC secretions contain a lot of growth factors that are important for regenerating damaged tissues (25). Despite the benefits of MSC transplantation, using secretions from these cells has more advantages than using the cells themselves. CM containing growth factors secreted from stem cells can be manufactured, freeze-dried, packaged, and transported more easily. Moreover, there is no requirement for donor-recipient matching to avoid transplant rejection problems or to worry about the possibility of malignancy (25, 42). The use of CM of mesenchymal stem cells or other cells instead of the cells themselves has also been used in the regeneration of various tissues. A study showed that CM transplantation of adipose stem cells could repair liver tissue in mice (26). Moreover, Khanmohammadi *et al.* reported that transplantation of BMSC-CM improved ovarian damage following chemotherapy by decreasing apoptosis in granulosa cells (13). Researchers have demonstrated that Schwann cell-CM promotes rat sciatic nerve regeneration after crush injury by increasing angiogenesis (43).

Among the limitations of this study was that the number of samples used was small and BMSC secretions in the CM were not separated and identified. Also, more molecular research is needed to clarify the mechanisms of peripheral nerve repair using MSC secretions.

## Conclusion

Since injection of BMSCs and BMSC secretions into the damaged sciatic nerve produced almost similar regenerative results, using these secretions may be an appropriate way to overcome the limitations of MSC transplantation for peripheral nerve injuries. The results suggest that BMSC secretions may improve remyelination and regeneration of damaged sciatic nerve by increasing the expression of MPZ and NGF and decreasing apoptosis.

## Authors’ Contributions

M G, A T, and P H performed data processing and collection and experiments; M A and N A analyzed and interpreted the results; A G, B Y, and M M critically revised and edited the article; SZ conceived, designed, supervised, directed, and managed the study; M G, A T, A G, P H, B Y, M M, M A, N A, and S Z approved the final version to be published.

## Conflicts of Interest

The authors have no conflicts of interest to declare.
